# Yoga for Neurodegenerative Disorders: Therapeutic Effects, Mechanisms, and Applications in Alzheimer’s and Parkinson’s Disease

**DOI:** 10.3390/sports13120458

**Published:** 2025-12-18

**Authors:** Federico Zoila, Maria Ida de Stefano, Alessia Sgobbio, Maria Antonietta Panaro, Angela Bruna Maffione, Laura Antonucci, Tarek Benameur, Michele Massaro, Socorro Vanesca Frota Gaban, Francesca Martina Filannino, Chiara Porro

**Affiliations:** 1 Department of Clinical and Experimental Medicine, University of Foggia, I-71100 Foggia, Italy; 2 Department of Neurosciences, Biomedicine and Movement Sciences, University of Verona, I-37134 Verona, Italy; 3 Department of Biosciences, Biotechnologies and Environment, University of Bari, I-70121 Bari, Italy; 4 Department of Biomedical Sciences, College of Medicine, King Faisal University, Al Hofuf 31982, Saudi Arabia; 5 Department of Food Engineering, Federal University of Ceara, Campus do Pici, Bloco 858, Fortaleza 60440-900, CE, Brazil

**Keywords:** motor function, cognitive function, neuroplasticity, oxidative stress, aging, neuroprotection, active aging, mind–body exercise

## Abstract

Neurodegenerative diseases (NDs) such as Alzheimer’s disease (AD) and Parkinson’s disease (PD) represent a growing global health concern with no definitive cure. Increasing evidence suggests that mind–body practices like yoga may offer neuroprotective benefits by modulating stress, neuroinflammation, and neuroplasticity. This narrative review explores the clinical outcomes, mechanistic insights, and biomarker evidence supporting yoga as a therapeutic intervention for AD and PD. Different studies indicate that regular yoga improves motor and cognitive functions, mood, and quality of life in affected individuals. At the molecular level, yoga enhances neurotrophic factors such as brain-derived neurotrophic factor (BDNF), reduces pro-inflammatory cytokines (e.g., IL-6, TNF-α), mitigates oxidative stress, and may preserve gray matter volume in key brain regions. These findings support the hypothesis that yoga induces favorable neuroplastic adaptations that may slow neurodegeneration. Despite encouraging early results, heterogeneity in study design, intervention duration, and sample size issues have limited the incorporation of neuroimaging and biomarker endpoints, which means further studies are warranted to clarify yoga’s therapeutic potential and mechanism in ND management.

## 1. Introduction

As life expectancy increases, the number of older people is increasing rapidly. This change increases risk factors for developing diseases related to the aging process, including NDs. The global prevalence of dementia is expected to double every twenty years, with an estimated 35.6 million people living with dementia in 2010, 65.7 million in 2030, and the number of people with dementia increasing rapidly to 115.4 million in 2050 [1]. These changes lead to chronic, low-grade inflammatory changes, impairing homeostasis, the pathogenic hallmark of such lifestyle disorders [2]. Moreover, lifestyle and dietary habits also increase the risk of NDs such as PD and AD [3].

The nature of NDs, the prevalence of the disease in the elderly, and the direct effects on the nervous system suggest that both the mind and body should be exercised from youth to old age. Furthermore, the promotion of physical education in schools is crucial, with studies highlighting its value for inclusive learning and improving sports methodology for young athletes [4,5]. Exercise is an essential component of ND treatment, as physical activity has been shown to reduce oxidative stress, slow motor decline, and improve mood impairments. However, traditional aerobic or resistance-based exercise requires safety monitoring, and some exercises are equipment-dependent [6].

Mind–body therapies are a form of complementary and alternative medicine. The National Center for Complementary and Alternative Medicine defines mind–body medicine as techniques that seek to “enhance the mind’s ability to affect bodily functions and symptoms” [7]. Mind–body therapies focus on the relationships between the brain, mind, body, and behavior and their impact on health and disease. These practices often provide stress relief and relaxation. Stress can exacerbate neurological disorders; therefore, adults use mind–body therapies more frequently to treat common neurological disorders than adults without neurological disorders, and more often when conventional treatments are deemed ineffective. Among mind–body therapies, yoga, breathing exercises, and meditation are the most used [8]. Mind–body practices can modify their emphasis and incorporate both mental and physical activity. While some, including mindfulness, meditation, and relaxation practices, focus on breathing and stress reduction, others, like yoga, tai chi, and qigong, are more exercise-concentrated [9].

Yoga is a comprehensive mind–body practice that originated 5000 years ago [10]. It offers well-documented physical, mental, and emotional benefits. It has also been shown to significantly improve measures of gait, flexibility, muscle force, fatigue, and quality of life in healthy elderly individuals and people with medical disorders, including back pain, arthritis, hypertension, anxiety, and depression [11].

The Sanskrit root yuj, which means “to yoke,” “to join,” or “to unite,” is where the word “yoga” comes from. It emphasizes the combination of body, mind, and spirit and represents the unification of the individual self with universal consciousness [9]. Yoga, which has its roots in ancient Indian philosophy, uses physical postures (asanas), breathing exercises (pranayama), and meditation (dhyana) to achieve balance and harmony. Together, these practices help with spiritual development and self-realization [10]. Due to its many physical, mental, and spiritual advantages, yoga has become increasingly well-known worldwide. Yoga, initially created to achieve spiritual enlightenment, provides an essential path towards inner calm and personal development [9]. Yoga is a multifaceted system with many variations that emphasize different physical, mental, and spiritual qualities rather than being a single, monolithic practice. Yoga has developed into several schools and styles, each with distinct practices and philosophies. Hatha yoga, a traditional style rooted in ancient India, stresses using dhyana, pranayama, and asanas to balance the body and mind [12]. The Sanskrit terms ha (meaning “sun”) and tha (meaning “moon”) are the origins of the word “Hatha”, which represents the harmony of opposing forces, including the sun and the moon, the masculine and feminine, and the active and receptive energies [12]. Through these exercises, hatha yoga seeks to promote balance and overall health. Yoga is available in various forms and offers multiple physical, mental, emotional, and spiritual benefits. It suits a wide range of audiences with different needs and preferences. A growing body of scientific evidence supports the many health benefits of yoga and highlights its value as a holistic practice that enhances overall well-being [13].

Regular yoga exercise leads to improved health and well-being. In Western cultures, yoga and meditation have emerged as tools to reduce stress, improve well-being, and promote general health. In recent years, yoga has been the subject of research as a therapeutic measure to prevent or treat medical conditions such as stress, insomnia, obesity, anxiety, diabetes, hypertension, oxidative stress, glucose tolerance, dyslipidemia, ND, and coronary heart disease [14]. Practicing yoga asanas and pranayama helps control total serum cholesterol, LDL, VLDL, and triglycerides [14]. Regular physical yoga practice has been shown to enhance posture, muscular strength, and balance, as well as increase flexibility and joint range of motion [10]. Furthermore, several studies described the beneficial effect of yoga on metabolic diseases such as diabetes and metabolic syndrome [15]. Due to its gentle approach, yoga is a promising intervention for people with PD who may not be able to engage in vigorous exercise. The theoretical basis for yoga practice is that integrating mind and spirit during movement balances the body and promotes recovery [5]. Therefore, it can be uniquely tailored to address specific risk factors for falls in PD, including posture, freezing of gait, poor balance, and lower limb weakness. Finally, yoga can motivate patients and improve cognitive function through the proposed mechanism of improved neuroplasticity, thereby enabling patients to participate in social and spiritual activities [11]. Despite the well-known benefits of yoga in selected chronic disease populations, evidence of the effect of yoga in persons with PD is limited. Researchers are increasingly investigating the potential benefits of yoga for people at risk for or diagnosed with AD, exploring its effects on physical performance, cognitive function, mood, and quality of life [9]. There is scientific evidence that yoga interventions can affect biological levels of systemic biomarkers of neuroplasticity, including hormones, neurotransmitters, inflammatory markers, oxidative stress markers, DNA damage markers, and telomere metabolism markers [16].

The guiding research question of this narrative review was: What clinical and mechanistic evidence supports the use of yoga as a therapeutic exercise for individuals with AD and PD? The purpose of this review was to integrate current knowledge on yoga-based interventions in AD and PD. The objectives were to summarize clinical findings on motor, cognitive, and emotional outcomes; to describe the mechanisms proposed in the literature, including neuroplastic and neuroimmune pathways; and to identify conceptual and methodological gaps that require further research.

## 2. Methodological Approach

This review employs a narrative approach because the available literature is heterogeneous in terms of design, outcomes, and intervention characteristics. A narrative synthesis allows integration of clinical findings with theoretical and mechanistic frameworks relevant to neurodegeneration.

The theoretical mechanisms discussed in this review were selected based on themes recurrently reported in clinical and experimental studies on mind–body interventions. Priority was given to mechanisms relevant to AD and PD pathology, including inflammation, oxidative stress, autonomic regulation, and neurotrophic signaling.

We conducted a structured literature search to ensure comprehensive coverage. Articles were identified through PubMed, Scopus, and Google Scholar using combinations of the terms “yoga”, “exercise”, “Alzheimer’s disease”, “Parkinson’s disease”, “neurodegeneration”, “mind–body”, and “neuroplasticity”. Additional references were identified by screening the bibliographies of relevant papers. The selection was not based on predefined systematic criteria; instead, studies were included when they contributed conceptual, clinical, or mechanistic insight into the role of yoga in AD or PD.

Data from the selected studies were extracted narratively, focusing on the following aspects: sample characteristics, type and duration of the yoga intervention, outcomes assessed, and mechanistic or physiological findings when available. Because the purpose of the review was synthesis and conceptual integration, no quantitative pooling or standardized coding was applied.

Data synthesis followed a thematic, narrative approach. Clinical findings were grouped by condition (AD or PD) and by outcome category (motor, cognitive, emotional, or biological). Mechanistic findings were integrated across studies to identify consistent pathways influenced by yoga practice.

## 3. Neurodegenerative Diseases

The ND are a heterogeneous group of neurological conditions, and they are defined as the progressive pathological loss of neurons in specific neural circuits, caused by the progressive loss of selectively vulnerable populations of neurons [17,18]. These diseases are a common and growing cause of mortality and morbidity worldwide, particularly in the elderly. The diagnosis is critical, allowing for a more reliable prognosis, and is often the guide to specific treatment and management [19].

The most common ND include AD, PD, Huntington’s disease (HD), and amyotrophic lateral sclerosis (ALS). It has been observed that distinct ND, which develop in different brain sites and show different etiologies, have similar cellular and molecular processes [20]. The main processes involved in neurodegeneration are: misfolded protein, which induces protein accumulation in the cell body; aging, which involves epigenetic changes, telomere attrition; and neuroinflammation, which causes the production of pro-inflammatory factors and oxidative stress [21,22].

Aging is linked to the most common ND [23]. A United Nations report estimates that 1 in 11 cases of ND will occur in people over the age of 65 in 2019, and this will rise to an alarming 1 in 6 by the end of 2050 [24]. With aging comes a gradual decline in tissue and organ function, and the resulting decline in physical well-being increases the likelihood of developing several age-related diseases, including neurological disorders, osteoporosis, and cardiovascular disease [23]. Brain aging and neurodegeneration are irreversible processes associated with changes in the brain microenvironment, such as genomic instability, epigenetic modifications, and loss of proteostasis [25]. The exact mechanisms by which aging is associated with neurodegeneration have not been identified, although aging is known to be a significant risk factor for ND [26]. The hallmarks of aging can be categorized into three distinct groups: primary, antagonistic, and integrative. Primary hallmarks include genomic instability, epigenetic changes, telomere attrition, and loss of proteostasis [26]. Antagonistic hallmarks describe compensatory responses to the primary damage linked to the aging process. Integrative hallmarks represent the outcome of cumulative damage to the primary and antagonistic hallmarks, and include stem cell exhaustion and impaired intercellular communication [26].

Neuroinflammation, including microgliosis and astrogliosis, refers to the inflammatory response within the central nervous system (CNS) and is a pathological hallmark of ND [27]. This response is mediated by glial cells reacting to various stimuli, such as infection, trauma, toxins, or NDs. Neuroinflammation plays a protective role in the defense of brain function by responding to pathogens and repairing damage and signs of injury [28]. Following an acute stressor, the inflammatory process is usually resolved. However, failure to resolve inflammation can lead to chronic or exacerbated neuroinflammation, resulting in neuronal damage and conditions in AD, PD, and HD [29]. Chronic neuroinflammation can occur in ND due to danger signals, such as protein aggregates, misfolded proteins, damaged synapses, Ca2+, or mitochondrial Reactive oxygen species (ROS) [30,31,32]. Several aggregated NDD proteins have been found to induce microglial activation, including Aβ, tau, α-synuclein, PrP fibrils, and SOD1. Many studies have shown that microglia can be polarized into two distinct phenotypes, classified as M1 (pro-inflammatory) or M2 (anti-inflammatory). M1 phenotype microglia express the cell surface markers CD86 and CD68 and release inflammatory mediators [33]. Indeed, activated M1 microglia and astrocytes release several inflammatory mediators, including cytokines such as IL-1β, IL-6, IL-8, and TNFα, prostaglandins, especially COX-2, and inducible nitric oxide, which increases ROS production [33,34,35]. These signals amplify neuroinflammation by affecting synaptic function and inducing neuronal cell death over time, permeating the blood–brain barrier to allow macrophage infiltration. In contrast, M2 phenotype microglia express Ym1 and CD206, release IL-4, arginase1, and IL-10, and show neuroprotective effects. The nucleotide-binding oligomerisation domain-pyrin domain-containing protein 3 (NLRP3) inflammasome plays a key role in ND [35,36]. Activation of the NLRP3 inflammasome in microglia and astrocytes leads to increased generation of ROS, which in turn causes oxidative stress in neurons and glial cells, exacerbating neuronal dysfunction and neurodegeneration and contributing to the progression of NDs. Oxidative stress is another hallmark of ND that is seen frequently [37]. Imbalances in calcium levels or malfunctions in the mitochondria can trigger an increased release of free electrons that react with oxygen or nitrogen. ROS are a naturally occurring result of cellular respiration and are integral to healthy metabolism [38]. They are formed when an electron detaches from the electron transport chain and binds with oxygen, forming superoxide anions. ROS are toxic when accumulated in the cell, so there are complex mechanisms to dispose of them under normal conditions [39]. However, when regulatory processes are disrupted, leading to an accumulation of these species, they can cause oxidative modifications of major cellular macromolecules, including lipids, proteins, RNA, and DNA. In response to oxidative stress, cells initiate the expression of pro-inflammatory genes and transcription factors, such as IĸB/NFĸB, which in turn induce the expression of various inflammatory genes, including those encoding chemokines and cytokines, thus perpetuating the inflammatory response [17,23].

Among the most common ND are AD and PD, which, according to recent studies, may originate from the exact neurodegenerative mechanism in the early phase. This neurodegenerative phenomenon opens the way to a new hypothesis for the etiopathological classification, early diagnosis, and therapeutic strategies of AD and PD, which are different manifestations of the same disease: Neurodegenerative Elderly Syndrome (NES) [40].

NES develops in three sequential phases: the seeding phase, the compensatory phase, and the bifurcation phase.

The initial phases of NES (seeding phase) are characterized by weak dysfunctions of monoamine neurotransmitters (including serotonin and the catecholamines dopamine, adrenaline, and noradrenaline) and alpha-synuclein (αSyn) (a neuronal protein that regulates the trafficking of synaptic vesicles and the subsequent release of neurotransmitters), which although they determine the onset of AD or PD do not produce detectable symptoms. The intermediate or compensatory phase is a pre-clinical phase where symptoms have not yet appeared, precisely compensated by the homeostasis of the different concentrations of monoamines. Finally, the bifurcation phase determines the manifestation of the disease, based on the affected dopaminergic area: ventral tegmental area (VTA) or substantia nigra pars compacta (SNc) [41,42,43].

Some factors can trigger the initial neurodegenerative process and direct it towards the onset of AD or PD or even modify its trajectory during the last phase. Genetic [44,45], environmental, and lifestyle factors like dietary habits [46], exposure to alcohol [47,48], and cigarette smoking [49,50,51,52], composition of the gut microbiota [53,54,55,56,57,58,59,60] significantly influence initial neurodegenerative progression during the seeding mechanism.

The NES hypothesis offers the possibility to investigate the causes of the two upstream pathologies in more depth, in the early silent pre-clinical phases, representing a new research frontier for early diagnosis and subsequent new treatment strategies for patients.

### 3.1. Alzheimer’s Disease

According to the World Health Organization, AD is considered a growing public health concern globally. The disease was first diagnosed in 1907 by Alois Alzheimer [46], but despite scientific evolution and new research hypotheses, there are still no promising treatments capable of halting or modifying the disease. AD accounts for 80% of all dementia cases, affecting approximately 44 million people worldwide. Dementia is a progressive, acquired cognitive decline that significantly impacts activities of daily living, resulting in dependence, disability, and death. Subjective cognitive decline (SCD), accompanied by mild cognitive impairment (MCI), associated with cerebrovascular risk factors (CVRFs), is known to increase the risk of developing dementia and AD.

From an etiopathological point of view, AD generally develops on a sporadic basis. Still, there is a rare familial form of AD that occurs with the mutation of 3 specific genes, amyloid precursor protein (APP), presenilin 1 (PSEN1), and presenilin 2 (PSEN2), and the onset is almost early, between the ages of 30 and 50 years [47]. Approximately 70% of AD risk is due to genetic factors. The APOE gene has three variants, ε2, ε3, and ε4, which are the main risk factors for sporadic AD. Thanks to genomic sequencing, more than 20 risk genes involved in inflammatory recycling pathways, cholesterol metabolism, and endosomal vesicles have been identified and studied. Not a single gene generates a high risk, but the combination of these increases the prediction that the event may occur [48]. Recent studies have investigated even further through next-generation sequencing, discovering that there are genes at low frequency, but that even alone produce a higher risk and offer insight into disease development.

The female gender is more affected; in fact, it represents an intrinsic risk factor for the development of AD. In particular, females carrying the *APOE-ε4* allele show a double probability of developing AD compared to males [49]. This allelic variant leads to abnormal neurovascular functioning, blood pressure, and heart rate [50]. With advancing age, the onset of menopause and the subsequent reduction in circulating estradiol, which has neuroprotective activity, may lead to an exacerbation of cognitive decline [51].

The first cells attacked by neuropathology are the basal forebrain cholinergic neurons (bfCN), which exhibit the appearance of beta-amyloid (Aβ) plaques and neurofibrillary tangles [52,53,54].

According to Armstrong et al. [55], the cascade hypothesis of AD proposes that amyloid precursor protein (APP) metabolism is disrupted by mutation or interleukin (IL)-mediated injury stress response, leading to the disruption of the microtubule-stabilizing protein tau and resulting in the formation of neurofibrillary tangles. Thus, neuropil filaments, dystrophic neuritis, astrogliosis, microglial activation, and cerebral amyloid angiopathy are observed [56]. Consequently, the pathological process leads to neurodegeneration with loss of synapses and neurons, resulting in macroscopic atrophy.

Amyloid plaques are extracellular accumulations composed of abnormal folding of Aβ-amyloid, which is composed of 40 or 42 amino acids (Aβ40 and Aβ42), two byproducts of APP metabolism that develop in the isocortex first and then in subcortical structures. In contrast, neurofibrillary tangles are paired helical filaments made of hyperphosphorylated tau.

Tau pathology spreads to the allocortex of the medial temporal lobe (entorhinal cortex and hippocampus) before spreading to the association isocortex. The resulting cytotoxic effects inevitably result in cortical cell death [57,58] and dysfunctions in cholinergic neurotransmission of the cerebral cortex driven by deficits in other target areas involved in learning, memory, and emotional regulation (hippocampus and amygdala) [59,60,61], resulting in the destruction of cognitive functions [62]. Therefore, AD is characterized by progressive memory loss and deficits in other cognitive domains (language, visuospatial skills, and executive functions). In the early and middle stages of the disease, other symptoms such as depression and apathy appear. In later stages, motor disorders (dystonia, tremor, etc.) may also occur [63]. Several studies suggest alterations in key dopaminergic nuclei, such as the ventral tegmental area (VTA) and the substantia nigra pars compacta (SNc) [64,65,66]. Pathological changes in the mesocorticolimbic dopamine (DA) circuit contribute to cognitive and behavioral symptoms and occur early in the disease progression [41,43]. In contrast, impairments in the mesostriatal DA system are associated with the development of extrapyramidal motor deficits, which typically arise in the later stages of AD [67]. Disruptions in serotonin (5-HT) production and transmission may also play a role in the pathogenesis of AD [68,69,70,71]. Finally, the locus coeruleus (LC), a dorsal pontine nucleus responsible for synthesizing norepinephrine (NA), which is involved in attention, memory, and other cognitive functions, may exhibit early impairments during disease progression [72,73,74,75].

### 3.2. Parkinson’s Disease

PD is a complex progressive ND first described in 1817 by James Parkinson [76]. The incidence of PD ranges from 5/100,000 to over 35/100,000 new cases per year [77]. The prevalence of PD increases with age, and the disease will be dramatically doubling in the following two decades, accompanied by increased health expenditure [78]. According to the latest data, early preventive treatments and care have become necessary and urgent, with the intention of reducing the incidence and the social-economic burden [79].

Pathologically, PD is characterized by a progressive degeneration of dopaminergic neurons in the substantia nigra pars compacta (SN), which participate in critical biological processes such as movement, motivation, and cognition. The progressive death of these neurons in the midbrain causes the typical motor symptoms of this disease. Another noted feature is the appearance of Lewy bodies and Lewy neurites, intraneuronal protein cytoplasmic inclusions, formed by insoluble alpha-synuclein clusters [80]. However, pathology also involves other brain regions. Clinical signs and symptoms are crucial for the diagnosis, especially the early disturbances that develop in the early stages of the disease, namely anosmia, depression, REM sleep disturbances, followed by motor deficits such as bradykinesia, slow progressive asymmetric resting tremor, and cogwheel rigidity. In the later stages of the disease, non-motor dysfunctions such as autonomic dysfunction, pain, and cognitive decline also appear [81]. Like AD, PD is a silent disease in the early stages. In fact, it has been validated that 50–70% of SN dopaminergic neurons are already damaged when motor disorders appear [82]. However, in the literature, more recent studies suggest that the loss of dopaminergic terminals in the basal ganglia determines the onset of motor deficits [83].

The etiology is multifactorial, so the onset of the disease depends on the combined effect of genetic and environmental factors. About 5–10% of cases are attributable to genetic mutations; however, at the same time, an involvement of genetic and environmental factors is also identified. A study comparing concordance rates in monozygotic and dizygotic twins found that the heritability of PD is only 30%, suggesting that most of the risk is associated with environmental and behavioral factors [84].

From a genetic point of view, PD is framed as a rather complex disease. In a recent study, more than 70 loci were identified that are associated with an increased risk of developing PD [85]. Among these, many are close to genes implicated in the lysosomal-autophagy system and the immune system, responsible for the role of alpha-synuclein misfolding.

In particular, the *PARKIN* and *PINK1* genes are involved in mitophagic processes, resulting in the accumulation of dysfunctional mitochondria, causing early-onset autosomal recessive PD [86,87].

Furthermore, *PARKIN* regulates PGC-1alpha, a transcriptional regulator that controls gene expression in mitochondrial biogenesis [88]. Thus, a dysfunction of mitochondrial turnover (degradation and biogenesis) occurs in PD. Some studies suggest that toxic substances such as rotenone and 1-methyl-4-phenyl-1,2,3,6-tetrahydropyridine (MPTP) inhibit the mitochondrial complex that causes the death of dopaminergic neurons in the SN [89]. Approximately 1–2% of PD cases are due to other genetic mutations, such as the *LRRK2* gene mutation that causes autosomal dominant PD or mutations in the *GBA* gene associated with autosomal recessive Gaucher disease [90]. Recent studies also mention the neuroinflammatory process as a possible upstream trigger of alpha-synuclein aggregation and neurodegenerative processes [91]. Diseases such as type 2 diabetes mellitus and inflammatory bowel disease increase the risk of developing PD [92,93].

Moreover, genetics is influenced by environmental factors such as exposure to pesticides, metals, pathogens, skull injuries, lifestyle, and nutrition. Epigenetic factors may also contribute to the pathogenesis of PD [94]. New neuroprotective strategies are emerging, such as alpha-synuclein clearance [95]. Clinical trials are underway that directly destroy oligomeric alpha-synuclein aggregates using monoclonal antibodies or an active vaccine. Genetic tests are helpful in diagnosing the disease, although they are not currently widely used in clinical practice.

## 4. Potential Beneficial Effect of Yoga on AD and Cognitive Function

Although there are AD drugs that can improve cognitive function, they do not stop the formation of Aβ plaques and tau tangles and have some side effects, such as gastric and intestinal disturbances, loss of appetite, headaches, confusion, and dizziness [96]. New research frontiers focus on non-pharmacological treatments, using a holistic approach that includes regular exercise, mind–body interventions, dietary modifications, and cognitive stimulation [97].

Among these non-invasive and promising strategies in improving cognitive and emotional health in AD patients, yoga stands out, an ancient mind–body discipline, whose effects have been widely studied in improving psycho-physical well-being, reducing depression, and above all, preventing the onset of AD in the elderly population [98].

Recent studies have shown that yoga practice modulates the levels of essential neurochemicals, such as BDNF, an afferent protein in the family of neurotrophins, molecules that protect the survival of existing neurons and promote the differentiation of new neurons and synapses. This neuroplastic improvement is associated with significant reductions in the severity of stress, anxiety, depression, and the management of AD [16,98,99,100].

In a case–control study conducted by Kaushik et al. [97], 30 participants (18 males, 12 females) diagnosed with mild to moderate AD and aged 60 years or older were enrolled and subjected to a 12-week program that included specific breathing exercises and meditation for one hour per day, six times per week. Neurocognitive assessment before and after the intervention produced significant improvements in the quality of life of the enrolled patients with a reduction in the Geriatric Depression Scale score and an increase in the Montreal Cognitive Assessment score, particularly in language, attention span, delayed recall, orientation, and visuospatial abilities. These preliminary findings offer valuable input into the search for complementary therapies to pharmacological therapy, as a therapeutic intervention in individuals with AD or who already present early signs of cognitive impairment, such as MCI [101], but larger studies are needed to investigate the long-term effects.

As mentioned previously, female gender, SCD, and CVRFs are known risk factors for AD. Yoga practice and meditation exercises positively impact stress, depression, inflammation, and cellular senescence. In this regard, a randomized controlled trial conducted on older adults with MCI demonstrated that Kundalini yoga (KY) exercises performed for 12 weeks had beneficial effects on cognitive function (memory and executive functioning) and mood (depression, apathy, and resilience) compared to memory enhancement training (MET) [102].

In a randomized, controlled trial, Grzenda et al. [103] investigated the effect of KY versus MET on memory performance at 12 and 24 weeks in 79 women at high risk for AD.

The enrolled population received 60 min in-person KY instruction with a certified instructor and guided exercises for the 12 min homework sequence. Whole blood samples were collected at T0, 12, and 24 weeks of follow-up for RNA sequencing and cytokine/chemokine assays. After 24 weeks of follow-up, women who performed daily KY exercises showed significant improvements in cognitive impairment compared to MET. At the neurochemical level, the chemokine eotaxin-1, a marker of aging, increased over time in MET but not KY participants. In humans, increased levels of eotaxin-1 with age have been associated with cognitive deficits, particularly in episodic and semantic memory [103]. Because it can cross the blood–brain barrier, peripheral eotaxin-1 may have cytotoxic effects on CNS neurons.

At 12 and 24 weeks, KY participants showed downregulation of signaling-related cytokines IL-10 and IFN-gamma, which promote the production of nitric oxide synthase and reactive nitrogen intermediates, as well as TNF-alpha from microglia that generates microvessel inflammation [104] and whose increase is associated with subjective complaints [105]. In AD mouse models, chronic intrahippocampal IFN-gamma expression is associated with increased microglial activation and thus AD pathology severity.

In conclusion, the results showed significant improvements in subjective cognitive function and changes in inflammatory and neurobiological markers, suggesting that yoga may positively influence neuroinflammation and neuroplasticity. A randomized trial found that older women at risk for AD who underwent 12 weeks of KY and Kirtan Kriya (KK) practice could preserve gray matter volume in several brain areas, compared to MET. Yoga also led to improvements in anxiety and depression. However, it did not significantly affect memory or resilience, likely due to the intervention’s short duration and the participants’ initial good health. The results suggest that yoga practice, due to its multimodal nature, may have neuroprotective effects, particularly on the hippocampus, and represent a promising approach for brain and mental health in older women at risk of AD. However, further larger and longer-term studies are needed [1].

## 5. Potential Beneficial Effect of Yoga on PD

Therapies used to treat PD mainly use medications combined with routine rehabilitation training. These therapies aim to reduce clinical symptoms and improve the quality of life in patients with this disease. Several studies have shown that practicing yoga, as an adjuvant strategy, could slow the progression of PD and improve motor function. Unlike traditional aerobic or resistance-based exercises that require safety monitoring and exercise equipment, yoga combines sitting and standing postures with breathing techniques and meditation to promote health and well-being [106].

Dyskinesia is a common complication in patients with PD and is very debilitating. The molecular mechanisms underlying dyskinesia involve complex alterations in dopamine receptor signaling and neuroplasticity in the CNS itself. Specifically, dysregulation of the cAMP/PKA signaling pathway and increased glutamate neurotransmission facilitate excessive and uncontrolled motor output. Furthermore, neuroplastic changes in dyskinesia are associated with altered expression and function of several neurotransmitter systems, such as the serotonergic system, which compensates for the loss of dopamine. This further complicates the synaptic environment and exacerbates the dysmodulation of striatal output pathways [107]. Typical motor symptoms in people with PD include tremor, muscle hypertonia, and bradykinesia. Numerous studies suggest that yoga can improve dyskinesia and daily activities in people with PD. Iyengar yoga, which provides relaxation exercises, yoga postures, and meditation, combined with conventional treatments, has significantly improved lower limb muscle strength, range of motion, and flexibility in patients with PD [108]. Furthermore, power yoga has been shown to reduce patient symptoms such as bradykinesia and stiffness in the upper and lower limbs while increasing lower limb muscle strength [109]. Hatha yoga has been studied as a therapy that affects gait and balance in patients diagnosed with PD. People with PD experienced a significant decrease in standing sway after yoga training and, consequently, improved static balance [110].

In the study by Swink et al., they showed that it is possible to combine yoga and occupational therapy for PD, Merging Yoga and Occupational Therapy, and that this would make a difference to the program. The 14 MY-OT sessions for PD were held over eight weeks. Significant improvements in balance were seen during the program, increasing body awareness during the sessions and helping to prevent falls in everyday life [111].

The 2024 study by Mailankody et al. [112] analyzed changes in patients with PD at three different time points: at the beginning of the study, after one month of conventional care, and after one month of supervised yoga sessions. Patients’ postural stability improved significantly after yoga.

Yoga intervention can significantly improve postural stability in patients with PD. A significant reduction in silent periods to cortical stimulation and short-interval intracortical inhibition suggests a decrease in GABAergic neurotransmission after yoga therapy, which may underlie the observed improvement in postural stability.

In patients with PD, a progressive reduction in bodily movements and physical functions occurs due to loss of physical strength and deterioration of neural motor function, affecting quality of life. One strategy that can compensate for the loss of automatic activities related to neural coordination is redirecting motor nerve commands through higher areas of the cerebral cortex [113]. A 2023 study found that practicing a combination of yoga asanas (physical exercises) and pranayama (breathing exercises) helps improve and maintain a balance between various physiological functions. Not only does the practice help relieve stress, but it also helps improve memory and comprehension skills. This was demonstrated by the statistically significant improvement in plaque and gingival indices from the first to the sixth month after yoga classes in patients with PD, where the yoga exercises contributed to improved toothbrushing ability [114].

A study by Kwok et al. showed that a group of patients with idiopathic PD who performed mindfulness meditation and yoga for 8 weeks had secondary outcomes, including improvements in motor and non-motor symptoms and serum interleukin-6 levels. Substantial evidence indicates that peripheral inflammation plays a role in the pathogenesis and prognosis of PD. In particular, pro-inflammatory cytokines such as IL-6, TNFα, and IL-1β can modulate signaling pathways, induce oxidative stress, disrupt neuronal function, and contribute to neurodegeneration [115].

Anxiety and depression directly impact the mental health of people with PD and can worsen the movement disorders associated with the disease. Yoga training involves practices, including breathing regulation and meditation, which can improve PD patients’ physical and mental health and reduce stress. One study found that meditation improved sleep disturbances and maintained a stable mood in nearly 40% of people. The exact mechanism by which yoga improves mood disorders in PD patients is unclear [116].

People living with PD often experience poor psychosocial well-being. Mobility issues, speech difficulties, and the stigma associated with the condition can lead to social withdrawal, exacerbating feelings of loneliness, social isolation, and depression. Yoga is a popular group activity within the PD community. It leverages cardiovascular fitness and controlled breathing, contributing to well-being by reducing stress and promoting parasympathetic nervous system dominance. Furthermore, yoga and group singing are both social activities involving movement in a group setting.

Good et al.’s study compared group singing and yoga, highlighting that both improved mood and decreased cortisol levels, which were used as biomarkers. This aligns with research suggesting that group activities reduce stress. However, it is noteworthy that only group singing elicited increased oxytocin, a key biomarker associated with social bonding. This is a departure from previous research, which also found this biomarker to increase after yoga training. One possible explanation for these discrepancies is that the initial stress of a new environment can influence neurohormonal responses. Reductions in cortisol and increases in oxytocin become more likely as participants become more comfortable over time [117].

## 6. General Effects of Yoga on Neurodegenerative Diseases

Integrating yoga as a non-pharmacological intervention in managing ND has attracted increasing scientific interest over the past decade. Since ND such as PD, AD, and MCI share overlapping pathophysiological mechanisms, including neuroinflammation, oxidative stress, and neuronal loss, interventions targeting holistic mind–body balance may offer cross-cutting therapeutic value. Yoga, as a multidimensional practice that includes postures (asana), breath regulation (pranayama), and meditative awareness (dhyana), is hypothesized to modulate neuroplasticity, stress reactivity, and immune function, mechanisms implicated in the progression of ND. Following, we synthesize the current literature on the general application of yoga in ND, highlighting its strengths, limitations, and future methodological research directions. The main studies investigating yoga-based investigating yoga-based interventions in AD and related neurodegenerative conditions are summarized in Table 1.

Although disease-specific studies dominate the literature, an emerging body of research evaluates yoga as a generalized intervention for cognitive decline and neurodegenerative disorders. A systematic review by Gothe et al. found that yoga interventions positively impacted brain regions associated with memory and cognition, particularly in older adults at risk for cognitive decline, suggesting its relevance for various ND [13]. Similarly, work by Mooventhan and Nivethitha [8] synthesized evidence across various neurological disorders, including AD and PD, concluding that yoga may improve neurophysiological function through improved autonomic regulation and reduced neuroinflammation. In individuals with MCI, a precursor to several ND, including AD, a RCT by Farhang et al. [121] compared a yoga-based mindfulness intervention with a psychoeducational session. Preliminary results showed increased cognitive flexibility and reduced anxiety in the yoga group, supporting its preventive potential. Complementing these behavioral data, Krause-Sorio et al. [1] demonstrated that yoga prevents hippocampal gray matter atrophy in older women at risk for AD, highlighting its neuroprotective effects detectable by structural Magnetic Resonance Imaging (MRI). The benefits of yoga also extend to populations with motor ND. In patients with ALS, Ribeiro [122] reported improved cramp management through Iyengar yoga in a small case series, noting improvements in functional mobility and patient-reported pain. For people with multiple sclerosis (MS), yoga interventions improved quality of life, physical self-efficacy, and motivation to exercise, as demonstrated in studies by Fasczewski et al. [123] and Gunnersen et al. [124].

The therapeutic influence of yoga in neurodegenerative neurons is thought to arise from its ability to modulate central and peripheral mechanisms. Psychologically, yoga is consistently associated with reduced perceived stress, which may mediate physiological outcomes. These mechanistic pathways are supported by recent evidence showing that structured exercise interventions in older adults improve physiological resilience, reduce systemic inflammation, and enhance biomarkers of healthy aging [125]. Danucalov et al. [126,127] demonstrated that yoga and compassionate meditation reduced stress and improved quality of life in caregivers of patients with AD, indirectly benefiting patient care and potentially mitigating environmental stressors that exacerbate cognitive decline.

Biologically, yoga influences central molecular pathways in neurodegeneration. Studies by Tolahunase et al. and Mohammad et al. [14,16] provide evidence that yoga-based interventions modulate biomarkers of neuroplasticity, including BDNF, inflammatory cytokines, markers of oxidative stress, and even telomere length, an indicator of cellular aging. These biomarkers are increasingly recognized as modifiable factors in neuromuscular neuropathy progression. Hassan et al. [128,129] extended this mechanistic investigation to in vitro models, proposing that yoga-mimicking conditions could protect neural tissues from AD, like alterations, including β-amyloid toxicity and tau phosphorylation. Although these latter findings are preliminary and derived from model systems, they lend biological plausibility to the neuroprotective claims associated with yoga. The heterogeneity of yoga delivery styles and methods represents both a strength and a challenge in clinical translation. Yoga interventions in the reviewed studies ranged from traditional hatha yoga [12] to hybrids of power and mindfulness yoga [109,119]. Each style emphasizes distinct dimensions of practice, physical effort, breath work, or meditative stillness, which may engage different neural and systemic processes. Remote delivery of yoga has shown promise in increasing accessibility among neurodegenerative populations. Allende et al. and James-Palmer & Daneault [130,131] both reported high adherence and safety in tele-yoga programs for patients with AD and PD, even in populations with chronic pain or motor limitations. These findings are particularly relevant given the progressive disability associated with most patients with NDs.

## 7. Discussion

Several studies reviewed here demonstrate methodological rigor. Randomized, controlled trial designs are increasingly common [132,133], and several clinical trials incorporate active comparators (e.g., stretching, memory training), which control for nonspecific effects of attention or social interaction [132,133]. Neuroimaging endpoints, such as hippocampal connectivity [133] and structural MRI [1], offer objective and quantifiable indicators of intervention effectiveness. Meta-analyses and systematic reviews, such as those by Ban et al. (2021) and Suárez-Iglesias et al. (2022) [106,134], consolidate the results of RCTs, reporting statistically significant effects on motor function, balance, and psychological well-being in patients with diabetic neuropathy. These aggregated results provide a basis for future clinical recommendations and help identify intervention characteristics that produce the most robust results.

### 7.1. Methodological Limitations and Challenges

Despite these strengths, the literature on yoga and ND has notable limitations. First, many studies involve small sample sizes and are underpowered to detect subtle effects. Studies such as Boulgarides et al. (2014) and Hall et al. (2011) [120,135], although suggestive, involved fewer than 15 people. The lack of long-term follow-up in most studies further limits the assessment of the impact of yoga on disease progression.

Second, interventions are highly variable across studies. Differences in session length, frequency, instructor expertise, and inclusion (or not) of breathing exercises and meditation hinder comparing results across studies or replicating findings. For example, Elangovan et al. (2020) [110] observed that while balance improved with hatha yoga in PD patients, gait did not, a nuance that may be related to the protocol design.

Third, few studies address the dose–response relationship or optimal duration of intervention. While some studies demonstrate benefits after 8–12 weeks, others suggest that neurobiological changes may require more prolonged exposure. Mailankody et al. (2024) [112] note that a 4-week yoga intervention improved measures of cortical excitability in PD patients, but noted that longer durations are likely needed to capture clinical outcomes [112]. Finally, there is a dearth of mechanistic biomarkers in most studies. Although some, such as those by Tolahunase et al. (2022) and Krause-Sorio et al. (2018) [1,16], incorporate biological endpoints, most rely solely on self-reported measures or clinician-administered scales. Standardizing outcome measures and including biomarkers will be critical to validate yoga as a biologically active intervention.

### 7.2. Future Directions for Research

Should future research prioritize multicenter RCTs? With standardized intervention protocols to enhance yoga’s translational potential in ND care. Such studies should include diverse ND populations and stratify outcomes by disease stage, allowing for targeted recommendations. Integrating neuroimaging and molecular biomarkers will help elucidate underlying mechanisms and identify responsive patient subgroups. Additionally, comparative effectiveness research is needed to evaluate the efficacy of yoga versus conventional physical therapy, cognitive training, or pharmacological agents. Hybrid interventions, such as yoga combined with occupational therapy or dietary counseling, should also be explored, especially in early-stage patients, where lifestyle changes may have the most significant impact [111].

Finally, patient-centered outcomes, such as adherence, acceptability, and perceived empowerment, should be incorporated into study designs. As demonstrated by Van Puymbroeck et al. (2018) and Arasappa et al. (2021) [118,136], yoga is generally well tolerated and positively perceived by patients, suggesting high feasibility in the clinical setting.

## 8. Conclusions

Yoga represents a promising, affordable, accessible intervention with potential benefits for ND. Modifying stress responses, inflammatory pathways, and neuroplasticity may slow functional decline and improve quality of life. Although preliminary results are encouraging, further high-quality research is necessary to establish standardized protocols, elucidate mechanisms, and determine the long-term efficacy. As the incidence of ND continues to increase globally, integrative approaches such as yoga should be considered as part of a comprehensive therapeutic strategy.

## Figures and Tables

**Table 1:** Detailed Methodological Matrix and Sample Characteristics of Included Randomized Controlled Trials (RCTs). The table summarizes the study design, intervention protocol, population size and specific conditions, and primary outcomes evaluated across the selected yoga-based intervention trials.

Ref. (Author, Year)	Method/Study Design	Evaluation (Design/Follow-Up)	Process (Protocol)	Sample Size and Characteristics (N, Age, Condition)	Variables Evaluated (Outcomes)	Generalization/Setting	Treatment (Type of Yoga)	Focus (Primary Goal)
Van Puymbroeck et al., 2018 [118]	RCT, Wait-list Controlled Pilot	Pre–Post (8 weeks)	Duration: 8 weeks. Freq.: 2x/week. Sess. Len.: 60–75 min.	N = 27 (15 Yoga/12 Control). PD, community-dwelling, with fear of falling.	Motor Function, Functional Gait, Postural Stability, Fall Risk (UPDRS, FGA, TUG).	Moderate (Community setting, focused on fall-risk PD).	Adaptive Therapeutic Yoga.	Improvement of functional mobility and reduction of fall risk.
Sharma et al., 2015 [108]	RCT, Controlled Pilot Study	Pre–Post (12 weeks)	Duration: 12 weeks. Freq.: 2x/week. Sess. Len.: 60 min.	N = 20 (10 Yoga/10 Control). PD (H&Y Stage I–III), mean age 67 years.	Motor Function (UPDRS Motor, BBS), Quality of Life (PDQ-39).	Low (Pilot study). Hospital-based clinic setting.	Hatha Yoga modified for PD.	Effect on motor function (bradykinesia, balance) and Quality of Life.
Kwok et al., 2019 [119]	RCT, Active Controlled	Pre–Post (8 weeks) and 12-week follow-up	Duration: 8 weeks. Freq.: 1x/week. Sess. Len.: 90 min.	N = 138 (69 Yoga/69 SRTE Control). Mild-to-moderate PD. Age 65 ± 8 years.	Psychospiritual Outcomes (Anxiety, Depression, Hardship/Equanimity) and HRQoL.	High (Large sample size for a yoga study). Community-based.	Mindfulness-based Yoga.	Psychospiritual outcomes and health-related Quality of Life (HRQoL).
Hall et al., 2011 [120]	RCT, Active Controlled (vs. Dance)	Pre–Post (12 weeks)	Duration: 12 weeks. Freq.: 2x/week. Sess. Len.: 60 min.	N = 50 (25 Yoga/25 Dance). Mild-to-moderate PD.	Balance, Gait, Freezing of Gait (FOG).	Moderate (Specific comparison group). Community-based rehabilitation setting.	Integral Yoga adapted for PD.	Comparison of Yoga vs. Dance therapy on balance and gait performance.
Elangovan et al., 2020 [110]	RCT, Non-blinded	Pre–Post (12 weeks)	Duration: 12 weeks. Freq.: 2x/week. Sess. Len.: 90 min.	N = 40 (20 Yoga/20 Control). Mild-to-moderate PD.	Cardiorespiratory Fitness, Muscle Strength, Flexibility.	Moderate (Clinic setting, focused on physical fitness).	Hatha Yoga modified for fall prevention.	Improvement of cardiorespiratory and physical fitness.
Farhang et al., 2022 [121]	RCT, Wait-list Controlled	Pre–Post (12 weeks)	Duration: 12 weeks. Freq.: 1x/week. Sess. Len.: 90 min.	N = 48 (24 Yoga/24 Control). Community-dwelling older adults, non-PD.	Health-Related QoL (SF-36), Mood (CES-D).	High (General elderly population).	Mindfulness-based Yoga (Hatha).	Effect on Quality of Life and Psychological Distress.
Ni et al., 2016 [109]	RCT, Controlled	Pre–Post (8 weeks)	Duration: 8 weeks. Freq.: 2x/week. Sess. Len.: 60 min.	N = 44 (22 Yoga/22 Control). PD patients (H&Y II-III).	Balance (BBS), Falls Efficacy (FES), Mobility (TUG).	Moderate. Community center.	Adaptive Hatha Yoga.	Improving balance and self-efficacy related to falls.
Kwok et al., 2022 [116]	RCT, Active Controlled (vs. Conventional exercise)	Pre–Post (12 weeks)	Duration: 12 weeks. Freq.: 3x/week. Sess. Len.: 60 min.	N = 80 (40 Yoga/40 Exercise). PD patients.	1 Quality (PSQI), Non-motor symptoms (NMSQ).	Moderate (Specific focus on non-motor symptoms).	Traditional Hatha Yoga.	Improvement of sleep quality and non-motor symptoms.
Kaushik et al., 2025 [97]	RCT, Wait-list Controlled	Pre–Post (12 weeks)	Duration: 12 weeks. Freq.: 3x/week. Sess. Len.: 60 min.	N = 52 (26 Yoga/26 Control). Mild-to-moderate AD.	Cognitive function (ADAS-Cog, MMSE), QoL.	Low (Early research stage for AD). Clinic setting.	Integrated Yoga (Asana, Pranayama, Meditation).	Effect on cognitive decline and Quality of Life in AD.
Swink et al., 2020 [111]	RCT, Wait-list Controlled	Pre–Post (8 weeks)	Duration: 8 weeks. Freq.: 2x/week. Sess. Len.: 60 min.	N = 34 (17 Yoga/17 Control). PD patients (H&Y I-III).	Balance (BBS), Mobility (TUG), Fear of Falling (FES).	Moderate. Rehabilitation center.	Modified Hatha Yoga.	Balance, mobility, and confidence in patients with PD.

Abbreviations: RCT, Randomized Controlled Trial; PD, Parkinson’s Disease; AD, Alzheimer’s Disease; H&Y Stage, Hoehn and Yahr Stage; UPDRS, Unified Parkinson’s Disease Rating Scale; BBS, Berg Balance Scale; TUG, Timed Up and Go; PDQ-39, Parkinson’s Disease Questionnaire-39; FGA, Functional Gait Assessment; FOG, Freezing of Gait; FES, Falls Efficacy Scale; HRQoL, Health-Related Quality of Life; NMSQ, Non-Motor Symptoms Questionnaire; PSQI, Pittsburgh Sleep Quality Index; ADAS-Cog, Alzheimer’s Disease Assessment Scale-Cognitive Subscale.

## Data Availability

The original contributions presented in this study are included in the article. Further inquiries can be directed to the corresponding author.
